# Dextran-Catechin: An anticancer chemically-modified natural compound targeting copper that attenuates neuroblastoma growth

**DOI:** 10.18632/oncotarget.10201

**Published:** 2016-06-21

**Authors:** Orazio Vittorio, Miriam Brandl, Giuseppe Cirillo, Kathleen Kimpton, Elizabeth Hinde, Katharina Gaus, Eugene Yee, Naresh Kumar, Hien Duong, Claudia Fleming, Michelle Haber, Murray Norris, Cyrille Boyer, Maria Kavallaris

**Affiliations:** ^1^ Children's Cancer Institute, Lowy Cancer Research Centre, UNSW Australia, Sydney, NSW, Australia; ^2^ Australian Centre for NanoMedicine and ARC Centre of Excellence in Convergent Bio-Nano Science and Technology, UNSW Australia, Sydney, NSW, Australia; ^3^ Department of Pharmacy Health and Nutritional Science, University of Calabria Arcavacata di Rende, Arcavacata, Rende CS, Italy; ^4^ ARC Centre of Excellence in Advanced Molecular Imaging, UNSW Australia, Sydney, NSW, Australia; ^5^ School of Chemistry, UNSW Australia, Sydney, NSW, Australia; ^6^ School of Chemical Engineering, UNSW Australia, Sydney, NSW, Australia; ^7^ Australian Centre for NanoMedicine, UNSW Australia, Sydney, NSW, Australia; ^8^ University of New South Wales Centre for Childhood Cancer Research, UNSW Australia, Sydney, NSW, Australia

**Keywords:** catechin, copper transporter, copper metabolism, childhood cancer

## Abstract

Neuroblastoma is frequently diagnosed at advanced stage disease and treatment includes high dose chemotherapy and surgery. Despite the use of aggressive therapy survival rates are poor and children that survive their disease experience long term side effects from their treatment, highlighting the need for effective and less toxic therapies. Catechin is a natural polyphenol with anti-cancer properties and limited side effects, however its mechanism of action is unknown. Here we report that Dextran-Catechin, a conjugated form of catechin that increases serum stability, is preferentially and markedly active against neuroblastoma cells having high levels of intracellular copper, without affecting non-malignant cells. Copper transporter 1 (CTR1) is the main transporter of copper in mammalian cells and it is upregulated in neuroblastoma. Functional studies showed that depletion of CTR1 expression reduced intracellular copper levels and led to a decrease in neuroblastoma cell sensitivity to Dextran-Catechin, implicating copper in the activity of this compound. Mechanistically, Dextran-Catechin was found to react with copper, inducing oxidative stress and decreasing glutathione levels, an intracellular antioxidant and regulator of copper homeostasis. *In vivo*, Dextran-Catechin significantly attenuated tumour growth in human xenograft and syngeneic models of neuroblastoma. Thus, Dextran-Catechin targets copper, inhibits tumour growth, and may be valuable in the treatment of aggressive neuroblastoma and other cancers dependent on copper for their growth.

## INTRODUCTION

Neuroblastoma is the third most common type of childhood cancer after leukemia and brain tumours. The majority of children are diagnosed with advanced stage disease and despite intensive therapy that includes highly toxic chemotherapy, surgery and bone marrow transplantation, neuroblastoma has survival rates of only 40–50% [1]. Moreover, due to the toxic side effects of chemotherapy, survivors of neuroblastoma frequently have lifelong health issues from the therapy they received as a child [2]. Therefore, targeted and less toxic therapies are urgently required to treat neuroblastoma and to improve the quality of life of the survivors.

Naturally occurring antioxidants have shown anticancer properties and often display low toxicity to normal cells [3]. Catechin is a natural antioxidant abundant in green tea that has demonstrated potential as a preventive agent for prostate and colorectal cancers [4–6]. Recently, Polyphenon E, a clinical grade mixture of green tea catechins under evaluation in multiple National Cancer Institute (Bethesda, MD) clinical trials, has been shown to have anticancer activity in a mouse model of neuroblastoma [7]. However, until now the low serum stability of catechin has limited its clinical application [8, 9]. To overcome the stability issue, we adopted a previously described bio-compatible chemical synthetic strategy for the conjugation of catechin with dextran [9]. This new conjugate, named Dextran-Catechin, showed increased serum stability compared to catechin alone, and anticancer activity in pancreatic ductal adenocarcinoma cells *in vitro* [10]. However, its broader effect in other aggressive cancers and drug resistant cells, as well as its mechanism of action are unknown.

In this study, we demonstrate that Dextran-Catechin is active against a panel of neuroblastoma cell lines harbouring clinically relevant genetic defects, including multidrug resistance. The efficacy of Dextran-Catechin was associated with elevated intracellular levels of copper in neuroblastoma cells. Our study revealed a novel mechanism of action for Dextran-Catechin that involves targeting copper homeostasis, induction of oxidative stress and apoptosis in neuroblastoma cells. Strong anticancer activity of Dextran-Catechin was shown in both human xenograft and syngeneic mouse models of neuroblastoma.

## RESULTS

### Dextran-Catechin reduces neuroblastoma cell viability

Since, the limited stability of catechin in serum remains one of the major challenges in its application, we used a bio-compatible synthetic strategy involving the conjugation of dextran with catechin to overcome serum stability issues (herein referred to as Dextran-Catechin). Dextran was selected because it is widely used in pharmaceutical synthesis; it is inexpensive, non-toxic and can be easily chemically modified. Our group has previously shown that dextran conjugation can increase the stability of chemotherapeutic agents and their penetration of the tumor mass [11]. Importantly, Dextran-Catechin showed activity and induced apoptosis in pancreatic ductal adenocarcinoma cells *in vitro* [10], however its broader activity in other aggressive cancers such as neuroblastoma, drug resistant cells and its mechanism of action have not been resolved.

**Figure 1:**
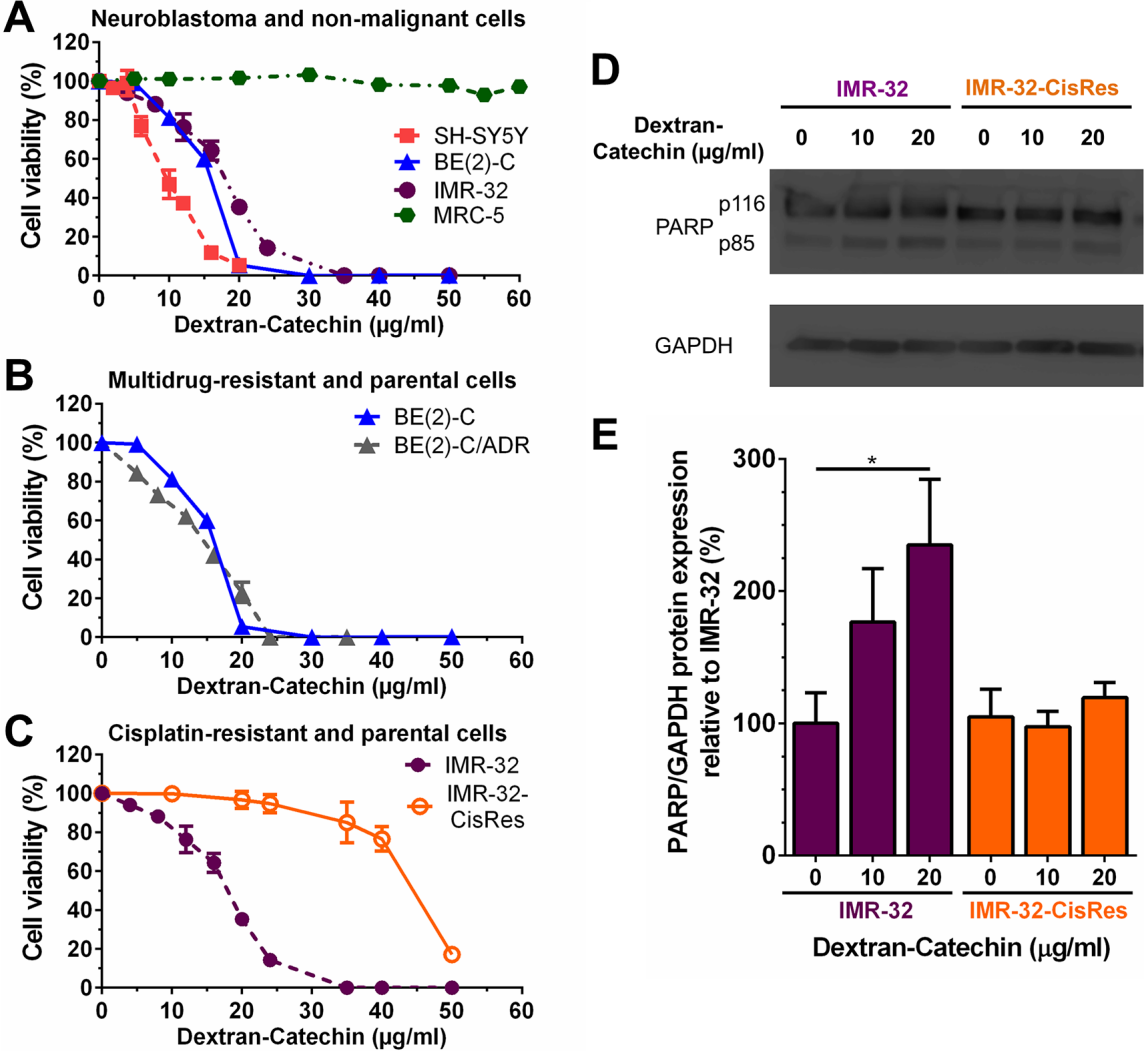
Effects of Dextran-Catechin on neuroblastoma cell viability (**A**) Neuroblastoma SH-SY5Y, BE(2)-C, and IMR-32 cells were highly sensitive to treatment with Dextran-Catechin for 72 h compared to non-malignant fibroblast MRC-5 cells. (**B**) Parental BE(2)-C cells and doxorubicin-resistant BE(2)-C/ADR cells treated with Dextran-Catechin for 72 h. (**C**) Cisplatin-resistant neuroblastoma IMR-32-CisRes cells were resistant to Dextran-Catechin compared to parental IMR-32 cells following treatment for 72 h. (**D**) Representative Western blots showing cleaved PARP in IMR-32 and IMR-32-CisRes whole-cell protein extracts following Dextran-Catechin treatment for 24 h. GAPDH expression was used as protein loading control. (**E**) Densitometry graph of Western blots showing increased expression of cleaved PARP in IMR-32 compared to IMR-32 CisRes cells treated with Dextran-Catechin. Values were normalized to GAPDH expression levels. *Columns*, means of at least three independent experiments; *Bars*, SEM (*p < 0.05).

Initially, we tested the effect of Dextran-Catechin on cell viability in a panel of neuroblastoma cells. Dextran-Catechin induced a strong reduction in neuroblastoma cell viability in three independent neuroblastoma cell lines, SH-SY5Y, BE(2)-C and IMR-32 (IC_50_: 9.7 ± 0.8 μg/ml, 16.0 ± 0.2 μg/ml and 17.83 ± 0.4 μg/ml respectively), following 72 h of treatment (Figure 1A). Importantly, when the effect of Dextran-Catechin was tested against non-malignant MRC-5 cells, no IC_50_ was reached even at doses as high as 60 μg/ml (Figure 1A). Moreover, no effect in neuroblastoma cells was observed with the individual components of catechin and dextran at doses up to 60 μg/ml (Supplementary Figure 1A and 1B). The IC_50_ for free Catechin was found to be 170 ± 3.84 μg/ml in IMR-32 (Supplementary Figure S2), which is about ten times higher than what we previously found for Dextran-Catechin (~17 μg/ml). This increased activity of Dextran-Catechin compare to the free Catechin is due to the improved stability of this conjugate in serum. We calculated a decrease of 60% for free Catechin after 72 h of incubation at 37°C in cell culture media containing 10% FBS serum. In sharp contrast, we observed an 8% decrease of Dextran-Catechin under the same conditions (Supplementary Data 3 and Supplementary Figure S3).

As drug resistance remains a major clinical problem in a range of cancers including neuroblastoma [12], we were interested in studying the effect of Dextran-Catechin in drug resistant cells. Firstly, the cytotoxic activity of Dextran-Catechin was investigated against neuroblastoma cells BE(2)-C selected for resistance to the anthracycline adriamycin (doxorubicin), BE(2)-C/ADR. These cells overexpress P-glycoprotein a well-characterised multidrug-resistance drug efflux pump, which has been shown to confer resistance to doxorubicin and other lipophilic anticancer drugs [13]. No resistance to Dextran-Catechin was observed in the BE(2)-C/ADR cells (IC_50_: 17.5 ± 0.2 μg/ml) compared to the parental BE(2)-C cell line (16.0 ± 0.6 μg/ml) (Figure 1B). These data suggests that Dextran-Catechin is unlikely to be effluxed by P-glycoprotein and that it may therefore represent a valuable drug conjugate for overcoming P-glycoprotein-mediated multidrug resistance.

Like doxorubicin, cisplatin is an important component of the combination chemotherapy for the treatment of neuroblastoma. Therefore, we investigated if Dextran-Catechin can by-pass cisplatin resistance in IMR-32 cells selected for resistance against cisplatin, and designated as IMR-32-CisRes cells (Supplementary Figure 4). Unexpectedly, IMR-32-CisRes cells were ~2.5-fold (IC_50_: 44.4 ± 0.8 μg/ml) more resistant to Dextran-Catechin compared to the parental IMR-32 cells (IC_50_: 17.83 ± 0.4 μg/ml) (Figure 1C). To further validate the effects of Dextran-Catechin in IMR-32 and IMR-32-CisRes cells, induction of apoptosis was examined by determining cleavage of PARP protein using western blotting following 24 h of treatment. A significant increase of cleaved PARP was observed in IMR-32 cells treated with 20 μg/ml Dextran-Catechin (Figure 1D). In marked contrast, no increase of cleaved PARP was observed in the IMR-32-CisRes cells at the same drug concentrations (Figure 1D). Collectively, these data demonstrate that Dextran-Catechin is less active in cisplatin-resistant neuroblastoma cells compared to the parental cells.

### Neuroblastoma cells with high intracellular copper levels are more sensitive to Dextran-Catechin

**Figure 2:**
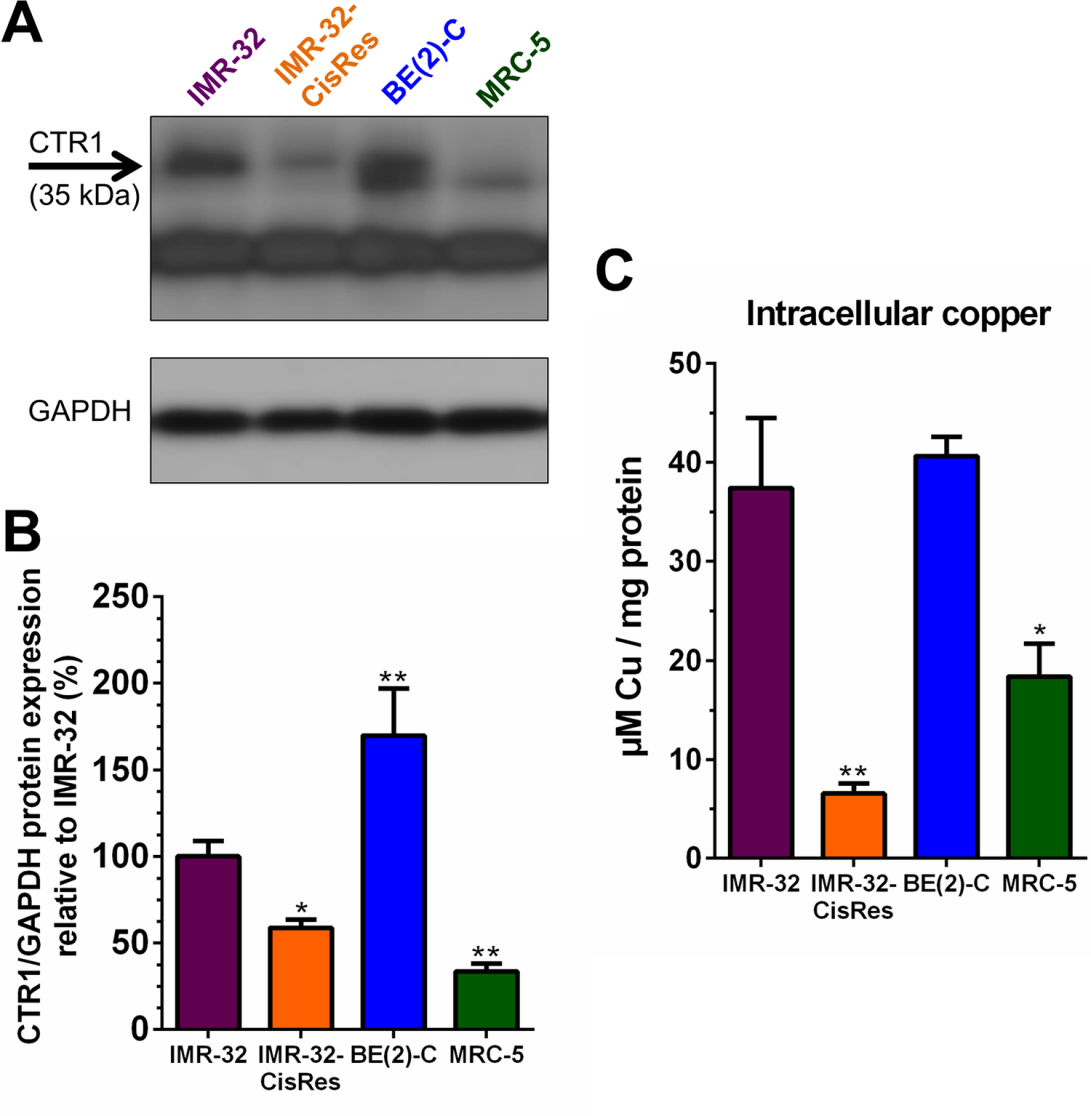
Levels of CTR1 protein expression and intracellular copper concentrations vary between neuroblastoma cell lines (**A**) Representative Western blot for CTR1 protein on whole-cell extracts from IMR-32, IMR-32-CisRes, BE(2)-C, and MRC-5 cells. GAPDH expression was used as a protein loading control. (**B**) Densitometry graph of Western blots showing higher expression of CTR1 in IMR-32 and BE(2)-C cells compared to IMR-32-CisRes and normal MRC-5 cells. Values are normalized to GAPDH protein expression and shown relative to CTR1 expression in IMR-32 cells (100%). (**C**) Intracellular copper levels are higher in IMR-32 and BE(2)-C cells compared to IMR-32-CisRes and MRC-5 cells. *Columns*, means of at least three independent experiments; *Bars*, SEM (*P < 0.05, **P < 0.01).

Identifying the basis for resistance to Dextran-Catechin in the cisplatin resistant neuroblastoma cells may lead to improved understanding on the mechanism of action of this compound in cancer cells. It has been reported that, in addition to its role in cellular copper uptake, CTR1 also functions as the main transporter for cisplatin and its down-regulation is correlated with cisplatin resistance [14]. To determine whether CTR1 levels differed between neuroblastoma drug resistant and sensitive cell lines, western blotting was performed. In the neuroblastoma IMR-32 and BE(2)-C cells that are sensitive to Dextran-Catechin (Figure 1A), the expression of CTR1 was significantly higher compared to that found in the MRC-5 cells that were not affected by the conjugate (Figure 2A and 2B). Interestingly, the cisplatin resistant IMR-32-CisRes cells that were cross-resistant to Dextran-Catechin had significantly lower levels of CTR1 compared to that of the parental IMR-32 cells (Figure 2A and 2B). Consistent with the protein data, similar differences in expression of *Ctr1* was confirmed at the gene expression level in IMR-32, BE(2)-C and MRC-5 cells (Supplementary Figure 5). However, unlike the protein data, the IMR-32 and IMR-32-CisRes cells expressed similar levels of the *Ctr1* gene (Supplementary Figure 5), suggesting the existence of a post-transcriptional regulatory mechanism in the resistant cells.

To investigate if there was a direct correlation between CTR1 expression and the intracellular copper levels a spectrophotometric assay was used. Results showed that BE(2)-C and IMR-32 cells had significantly higher intracellular copper levels compared to IMR-32-CisRes and MRC-5 cells (Figure 2C). Overall, these results indicate that high expression levels of CTR1 are associated with high intracellular copper, which in turn correlates with the cytotoxic activity of Dextran-Catechin in neuroblastoma cells.

### Depletion of CTR1 expression reduced intracellular copper levels and led to a decrease in neuroblastoma cell sensitivity to Dextran-Catechin

**Figure 3:**
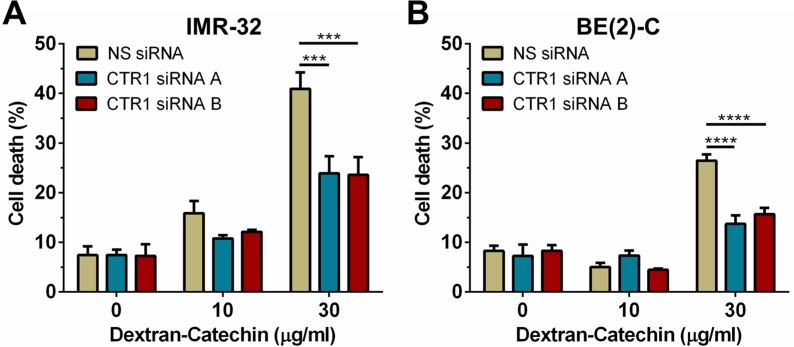
Knockdown of CTR1 in IMR-32 and BE(2)-C cells significantly reduced their sensitivity to Dextran-Catechin (**A**) Cell death in IMR-32 and (**B**) BE(2)-C cells after knockdown of CTR1 and subsequent treatment with Dextran-Catechin for 24 hours. *Columns*, means of at least three independent experiments; *Bars*, SEM (***p < 0.001, ****p < 0.0001).

Previous studies have shown that suppression of CTR1 with siRNA causes a reduction in intracellular copper levels [15]. To investigate if the expression level of CTR1 and consequent decrease of intracellular copper influenced the anticancer activity of Dextran-Catechin we partially suppressed the expression of CTR1 in neuroblastoma cells. Since copper is essential for cellular survival [16] the partial suppression of CTR1 was optimised to minimise effects on cell survival. Two different siRNAs, CTR1 siRNA A and CTR1 siRNA B, were optimised for suppression of CTR1 in IMR- 32 and BE(2)-C cells at the gene and protein levels (Supplementary Figure 6). Consistent with published data [15], depletion of CTR1 by siRNA caused a significant reduction of intracellular copper in neuroblastoma cells (Supplementary Figure 7). In order to determine the impact of CTR1 expression, and hence copper levels, on Dextran-Catechin activity, IMR-32 and BE(2)-C cells were transfected with a control non-silencing siRNA (NS siRNA) and two CTR1 independent siRNAs and 12 h later incubated with 10 and 30 μg/ml of Dextran-Catechin for 24 h prior to performing the trypan blue exclusion assay. In both CTR1 suppressed IMR-32 (Figure 3A) and BE(2)-C (Figure 3B) cells, the induction of cell death following 24 h of treatment with 30 μg/ml Dextran-Catechin was significantly lower compared to cells transfected with non-silencing siRNA (NS siRNA). These results show that the reduction of intracellular copper in neuroblastoma cells reduced sensitivity to Dextran-Catechin.

### Copper potentiates Dextran-Catechin activity in neuroblastoma cells

**Figure 4:**
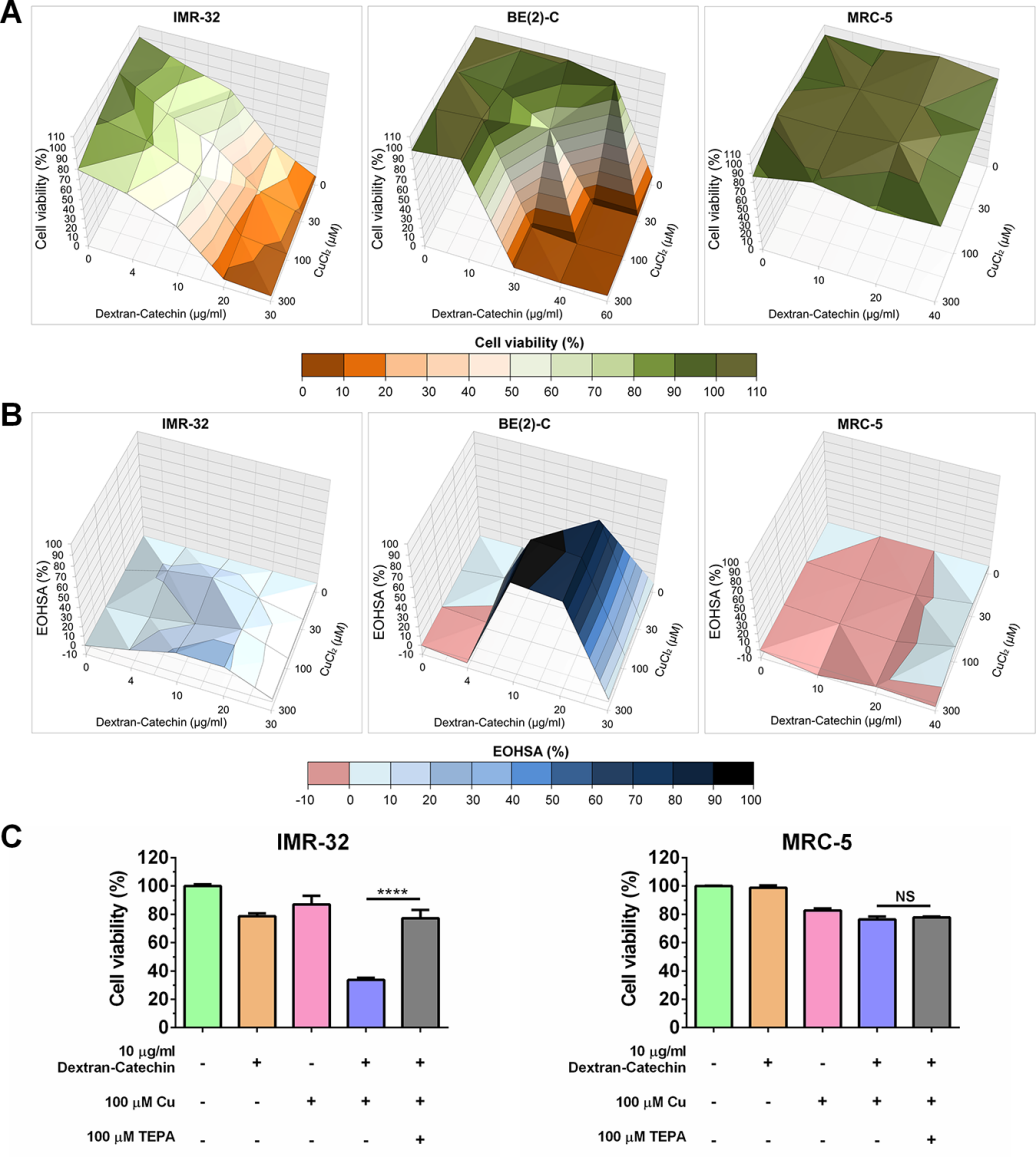
Copper potentiated Dextran-Catechin activity in neuroblastoma cells (**A**) Surface plots illustrating cell viability after combination treatment with Dextran-Catechin and CuCl_2_ for 48 h in IMR-32, BE-2(C) and MRC-5 cells. (**B**) Surface plots of the Excess Over Highest Single Agent (EOHSA) show the difference in cell viability inhibition between Dextran-Catechin and CuCl_2_ and the most potent single agent at corresponding dose in IMR-32, BE(2)-C and MRC-5 cells. Shades of blue colour-code positive values and thus represent greater effect of the combination treatment compared to single agents. (**C**) Viability of IMR-32 and MRC-5 cells after single or combination treatment with Dextran-Catechin and CuCl_2_, and/or depletion of copper by addition of the chelation agent tetraethylenepentamine (TEPA). *Columns*, means of at least three individual experiments; *Bars*, SEM (****p < 0.0001).

The finding in the preceding section that partial suppression of CTR1 led to reduced efficacy of Dextran-Catechin in neuroblastoma cells further highlights the link between the activity of this compound and copper. To further explore whether copper is important for the activity of Dextran-Catechin, we performed a combination treatment study. Cell viability of neuroblastoma cells IMR-32 and BE(2)-C, and non-malignant cells MRC-5 was measured after treatment with Dextran-Catechin and copper (CuCl_2_) alone or in combination at different concentrations. Combination treatment with Dextran-Catechin and copper led to higher inhibition of cell viability compared to either compound alone in neuroblastoma, but not in MRC-5 cells (Figure 4A). The combination effect was further assessed by determining the Excess Over Highest Single Agent [17] (EOHSA), which allowed for identification of combination dose pairs exceeding the most effective single agent at the corresponding concentration. In IMR-32 and BE(2)-C cells, combination treatment with Dextran-Catechin and copper exhibited a stronger reduction in cell viability compared to individual compounds for the majority of dose pairs (Figure 4B). In sharp contrast, copper did not potentiate the activity of Dextran-Catechin in non-malignant MRC-5 cells (Figure 4B).

To further validate the role of copper in Dextran-Catechin efficacy, we performed additional experiments that involved removing copper from the media by using the copper chelating agent tetraethylpentamine [18] (TEPA) (Figure 4C). In IMR-32 cells treated with Dextran-Catechin (10 μg/ml) only, viability decreased by ~20% in 48 h. When Dextran-Catechin (10 μg/ml) was added in combination with 100 μM CuCl_2,_ cell viability decreased by ~65% in 48 h (Figure 4C). Importantly, when IMR-32 cells were incubated with a combination of Dextran-Catechin (10 μg/ml), CuCl_2_ (100 μM) and TEPA (100 μM; equimolar concentration of copper), the higher activity obtained by adding copper to Dextran-Catechin was abrogated by the copper chelating agent, and the cell viability was similar to that observed for single treatment of Dextran-Catechin. In contrast in, MRC-5 cells that are not sensitive to Dextran-Catechin (Figure 1A) and that have low CTR1 expression and low intracellular copper (Figure 2B and 2C), the combination of Dextran-Catechin and CuCl_2_ in the media did not significantly affect cell viability (Figure 4C). These results provide further evidence that copper accumulation in neuroblastoma cells plays an important role in the anticancer activity of Dextran-Catechin. It has been previously demonstrated that an increase in copper in the extracellular environment or the inside of cells is associated with down-regulation of CTR1 to maintain copper homeostasis [19]. CTR1 is then negatively regulated by increasing intracellular copper levels. To validate this observation in our model we demonstrated the down-regulation of CTR1 in neuroblastoma IMR-32 cells after the supplementation of the cell culture media with copper (Supplementary Figure S9). Importantly, Dextran-Catechin was more effective in the presence of copper (Figure 4), even though this induced down-regulation of CTR1, clearly demonstrating that the activity of Dextran-Catechin is influenced by the copper concentration levels rather than the uptake of Dextran-Catechin by CTR1.

### Dextran-Catechin increases oxidative stress and impairs reduced glutathione in neuroblastoma cells

**Figure 5:**
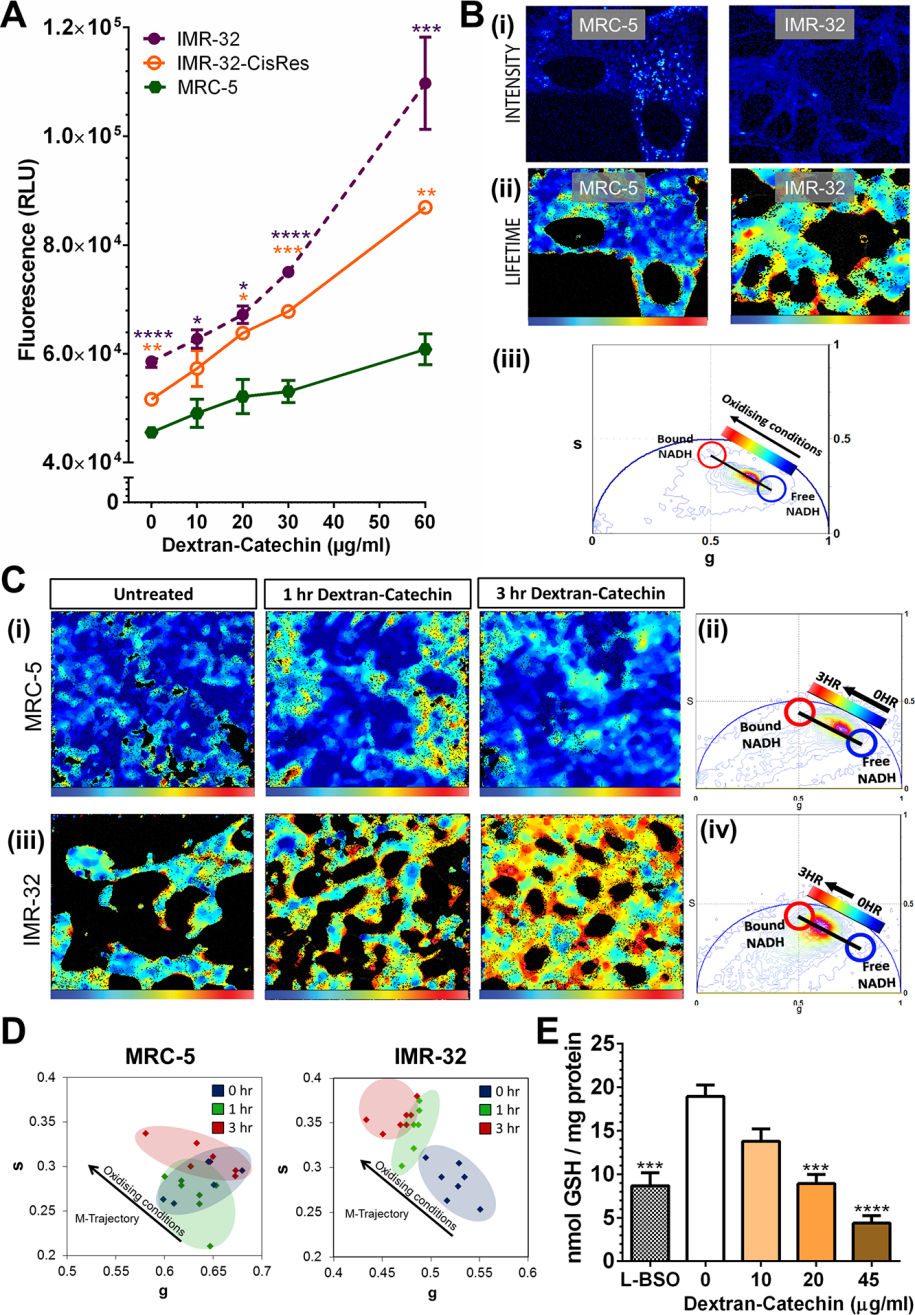
Dextran-Catechin treatment induced the generation of reactive oxygen species and the depletion of anti-oxidants in neuroblastoma cells (**A**) Quantitation of fluorescence intensity based on staining with CellROX^®^ Oxidative Stress Reagent in IMR-32, IMR-32-CisRes and MRC-5 cells after 5 h incubation with different concentrations of Dextran-Catechin. (**B**) (i) Two-photon fluorescence intensity image excited at 740 nm of MRC-5 and IMR-32 live cells and (B) (ii) Lifetime images of MRC-5 and IMR-32 pseudo-coloured according to free / bound NADH ratio. Blue colour indicates a high ratios of free / bound NADH, while cyan, yellow and red represent a linearly and progressively decrease in the free / bound NADH ratio (palette defined in (B) (iii)). The ratio of free / bound NADH indicates the level of oxidising conditions. (B) (iii) Phasor plot histogram with coordinates s and g showing the lifetime distribution of auto-fluorescence detected in each pixel from the intensity images of (B). The linearity of this distribution reflects the relative amount of free and bound NADH and a shift towards bound NADH is indicative of oxidising conditions. (**C**) (i) Lifetime images of the change in metabolism of MRC-5 cells upon administration of Dextran-Catechin 20μg/ml pseudo-coloured according to the free / bound NADH ratio defined in (C) (ii) The phasor plot. (C) (iii) Lifetime images of the change in metabolism of IMR-32 cells upon administration of Dextran-Catechin 20μg/ml pseudo-coloured according to free / bound NADH ratio defined in (C) (iv) The phasor plot. (**D**) Cell phasor of changes in oxidising conditions in cells treated with Dextran-Catechin. Oxidising conditions hardly changed in MRC-5 cells following treatment, while the NADH lifetime distribution in IMR-32 cells followed a metabolic trajectory (M-trajectory) from free to protein-bound NADH representing a shift towards oxidising condition, (*n* = 7). (**E**) Treatment with 10–45 μg/ml Dextran-Catechin for 3 h reduced cytoplasmic GSH levels compared to untreated IMR-32 cells. The inhibition induced by 3 h treatment with 45 μg/ml Dextran-Catechin exceeded that obtained by 24 h treatment with the GSH inhibitor L-BSO. *Columns*, means of at least three individual experiments; *Bars*, SEM (****P ≤ 0.0001, ***P ≤ 0.001, **P ≤ 0.01, *P ≤ 0.05).

Catechin is widely known as an antioxidant, however, in certain conditions, such as in the presence of high levels of metal ions, it can induce reactive oxidative stress species [20]. In particular, chemical studies have demonstrated that Cu^2+^ converts catechin from an antioxidant to a pro-oxidant [21]. However, the pro-oxidant activity of Dextran-Catechin in cancer cells has never been associated with their intracellular copper levels. In this study we have shown that neuroblastoma cells with high levels of intracellular copper are more sensitive to Dextran-Catechin (Figures 1 and 2). We therefore investigated whether the drug activity in these cells is mediated by the generation of oxidative stress using a commercial fluorogenic probe (CellROX^®^) designed to reliably measure reactive oxygen species in the cells. CellROX^®^ is a cell-permeable reagent that is non-fluorescent while in a reduced state and upon oxidation exhibits a strong fluorogenic signal [22]. We measured the intensity of oxidative stress by using a fluorometric microplate reader in IMR-32, IMR-32-CisRes and MRC-5 cells after 5 h of treatment with Dextran-Catechin. As anticipated, the generation of oxidative stress was stronger and increased rapidly in the IMR-32 cells (Figure 5A), which have higher intracellular copper compared to IMR-32-CisRes and MRC-5 (Figure 2B).

To further validate these data we used fluorescence lifetime imaging microscopy (FLIM), a sophisticated technique that allowed us to monitor the changes in oxidative state of single cells in response to Dextran-Catechin in real time. Here, we used the phasor approach to FLIM to assess the intracellular reduction-oxidation ratio by measuring NADH in the free and bound form [23, 24]. This technique has been previously validated in a study that identified oxidative stress induced by a respiratory chain inhibitor through a decrease of the effective lifetime of NADH (mean lifetime of free and protein-bound NADH) in cells [25]. FLIM phasor cluster analysis detects changes in the free / bound NADH ratio in each pixel of a FLIM image, and thus provided a spatial map illustrating the effect of Dextran-Catechin on levels of oxidative stress in cells [26]. Figure 5B (i) shows two-photon intensity images of cellular auto-fluorescence of MRC-5 and IMR-32 cells excited at 740 nm. Figure 5B (ii) shows FLIM maps pseudo-coloured according to free / bound NADH (phasor distribution shown in Figure 5B (iii)). Changes in the linear distribution of free / bound NADH ratio indicated that neuroblastoma IMR-32 cells have a different basal level of oxidative stress compared to non-malignant MRC-5 cells. The NADH lifetime distribution in MRC-5 was characterised by a higher free / bound NADH ratio compared to IMR-32 cells. In fact, IMR-32 cells had intrinsically more bound NADH and thus were under higher levels of oxidative stress than MRC-5 cells (Figure 5B (iii)). When MRC-5 cells were treated with Dextran-Catechin, lifetime images and phasor plot histograms showed initially a slight induction of oxidative stress (1 h of treatment), however, the cells recovered and oxidative stress decreased at the 3 h time point (Figure 5C (i) and Figure 5C (ii)). In sharp contrast, when IMR-32 cells were treated with Dextran-Catechin an incremental increase in oxidative stress from 0–3 h was observed (Figure 5C (iii) and Figure 5C (iv)). The linear distribution of free / bound NADH ratio illustrated in phasor plot histograms therefore shifted towards more bound NADH in IMR-32 following Dextran-Catechin treatment. In conclusion, these data showed that treatment with Dextran-Catechin did not change the free / bound NADH ratio in MRC-5 cells between 0 and 3 h, however, it induced a shift towards a lower free / bound NADH ratio over time in IMR-32 cells. The M-trajectory (metabolic trajectory) in Figure 5D illustrates this shift towards a lower free / bound NADH ratio and oxidising conditions in Dextran-Catechin treated IMR-32, which was not found in treated MRC-5 cells. Dextran-Catechin-induced oxidative stress was therefore specific to IMR-32 cells that have high intracellular copper. This is consistent with our chemical experiment showing that Dextran-Catechin generates reactive oxidative species in the presence of copper by Fenton reaction (Supplementary Data S8 and Supplementary Figure S8).

The main intracellular copper binding agent regulating copper uptake in cells is reduced glutathione (GSH) [27]. Importantly, GSH plays a major role in the maintenance of the intracellular redox balance, and is also involved in many metabolic processes and in drug resistance [28]. The binding of copper with GSH, limits the redox reaction of this metal ion with pro-oxidant agents such as Dextran-Catechin. GSH is thus a key component, which regulates intracellular copper levels and protects cancer cells from oxidative stress. To further investigate the mechanism of action of Dextran-Catechin, we studied whether this compound affects cytoplasmic GSH levels in neuroblastoma cells. Treatment with Dextran-Catechin resulted in a dose-dependent decrease in cellular GSH in IMR-32 cells (Figure 5E). Interestingly, Dextran-Catechin induced a stronger decrease of GSH compared to the potent GSH depletion agent L-BSO (positive control), an agent used to sensitize cancer cells to pro-oxidant chemotherapies [29]. Taken together these results show that Dextran-Catechin, not only induces strong oxidative stress in neuroblastoma cells (Figure 5A–5D), but also causes a significant reduction of GSH levels (Figure 5E), depleting this important anti-oxidant system and de-regulating cellular copper homeostasis.

### Anticancer activity of Dextran-Catechin in *in vivo* models of neuroblastoma

**Figure 6:**
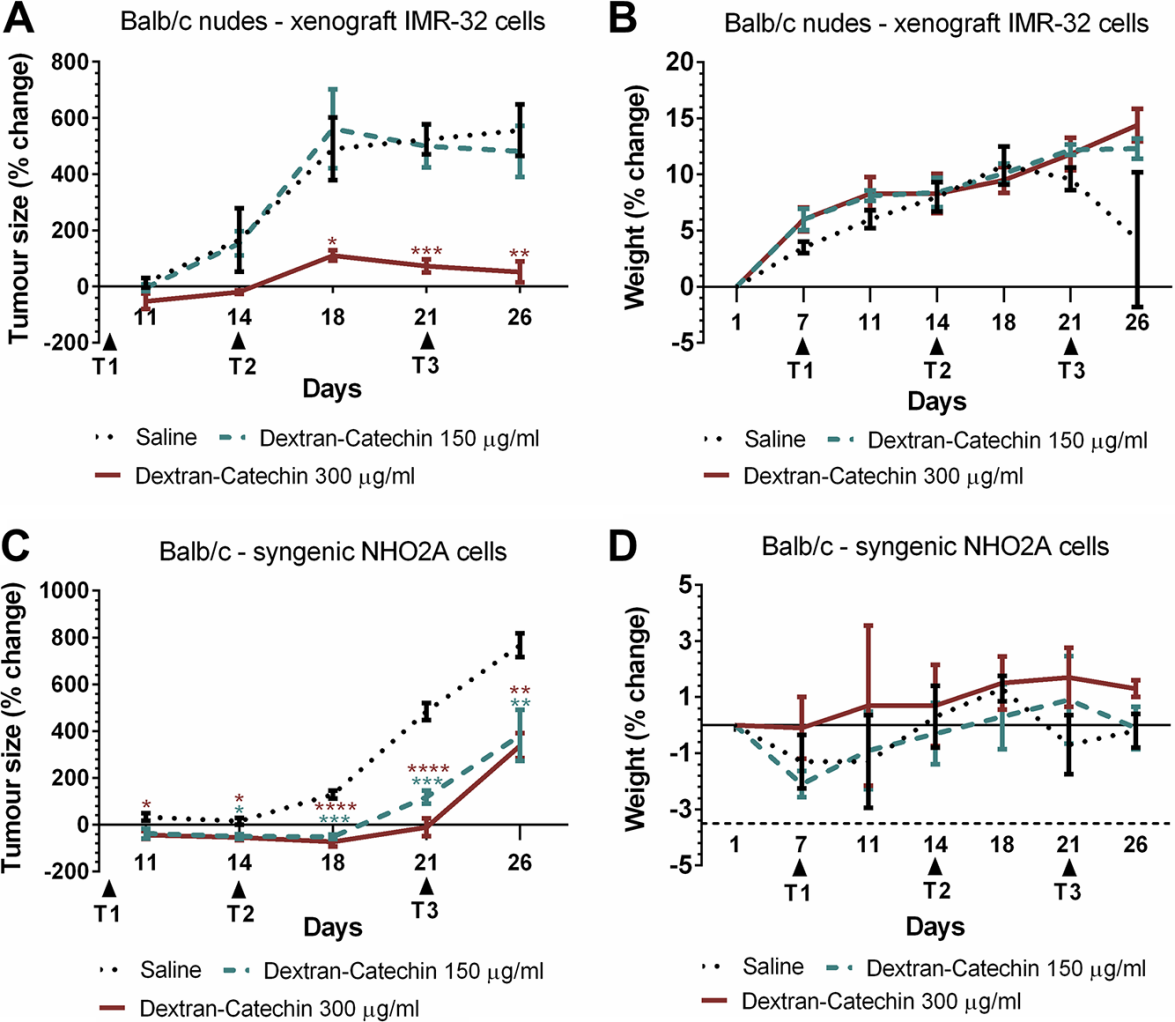
Dextran-Catechin showed significant anticancer activity in neuroblastoma mouse models (**A**) Nude mice (BALB/c) injected subcutaneously with human IMR-32 cells were treated with 150 or 300 μg/ml Dextran-Catechin when the mean tumour volume reached about 200 mm^3^. Treatment administered i.v. weekly for three weeks significantly reduced tumour growth in mice treated with 300 μg/ml Dextran-Catechin compared to the saline control cohort. (**B**) Weight of tumour bearing nude (BALB/c) mice for the saline control and Dextran-Catechin treated groups showed no significant difference. (**C**) Syngeneic neuroblastoma model using immuno-competent mice (BALB/c) injected subcutaneously with murine neuroblastoma NHO2A cells and treated with 150 or 300 μg/ml Dextran-Catechin when the mean tumour volume reached about 200 mm^3^. Treatment administered i.v. weekly for three weeks significantly reduced tumour growth in Dextran-Catechin treated mice compared to the saline control. (**D**) Change in (BALB/c) mouse weights for the saline control and Dextran-Catechin treated groups in the syngeneic neuroblastoma model showed no significant differences. *Columns*, means of at least three individual experiments; *Bars*, SEM (*p < 0.05, **p < 0.01, ***p < 0.001, ****p < 0.0001).

Although Dextran-Catechin has increased stability and efficacy compared to natural catechin *in vitro* [10], the *in vivo* anticancer activity of this conjugate has never been tested before. We therefore investigated the dose response to this conjugate in a human xenograft model and a mouse syngeneic model of neuroblastoma. These experiments aim to determine the dose-response relationship of Dextran-Catechin in a xenograft neuroblastoma model and to define if the compound affects animals' weight at the concentrations tested. The subcutaneous human neuroblastoma xenograft model was established using human IMR-32 cells. When tumours reached approximately 200 mm^3^ mice were treated weekly for three weeks with Dextran-Catechin (150 or 300 μg/ml) or saline control. Dextran-Catechin 300 μg/ml led to a significant reduction in tumour volume over the 26 day experimental period, compared to saline control (Figure 6A). No significant decrease with Dextran-Catechin at 150 μg/ml was observed. The strong anticancer activity demonstrated with 300 μg/ml Dextran-Catechin did not lead to adverse effects or a reduction of animal body weight (Figure 6B).

To investigate the anticancer potential of Dextran-Catechin in a mouse model with an intact immune system, a mouse syngeneic model of neuroblastoma was used. NHO2A cells have been derived from the transgenic TH-MYCN neuroblastoma mouse model that develops aggressive neuroblastoma and shows many features of the human childhood disease [30]. NHO2A cells were engrafted subcutaneously into immune-competent mice and tumours allowed to reach 200 mm^3^ before treatment commenced. Mice were treated weekly with Dextran-Catechin (150 or 300 μg/ml) or saline control for three weeks. An inhibition of the tumour volume was observed in the syngeneic neuroblastoma model at both 150 μg/ml and 300 μg/ml of Dextran-Catechin compared to saline-treated control mice (Figure 6C). However, in the syngeneic mouse model Dextran-Catechin was slightly less effective in reducing the tumour growth compared to the effect of the same dose administrated to the human xenograft model. The result in the syngeneic model is consistent with the low intracellular copper levels present in the NHO2A cells (Supplementary Figure 10A and 10B). Importantly, no significant adverse effects or a reduction in the body weight of the treated mice was observed during the treatment period (Figure 6D).

The strong *in vitro* and *in vivo* anticancer activity of Dextran-Catechin in neuroblastoma, its unique mechanism of action, combined with the low risk of side effects makes this modified natural compound a promising agent for the treatment of neuroblastoma and other cancers in which copper drives tumour growth.

## DISCUSSION

In traditional Chinese medicine green tea is believed to have health benefits [31] and two clinical phase II trials showed the preventive effects of green tea in patients with prostate intraepithelial neoplasia [6] and on high-risk oral premalignant lesions [32]. Catechin is a natural active ingredient of green tea responsible for the anticancer activity, but its low serum stability limits its clinical application. Herein, we have shown that a conjugate of this natural component, Dextran-Catechin, with improved serum stability is effective against neuroblastoma cells. Moreover, compelling evidence is provided showing that Dextran-Catechin targets copper metabolism and induces oxidative stress in neuroblastoma cells. Importantly, Dextran-Catechin was found to be effective against two independent neuroblastoma tumor models *in vivo* and may be a valuable therapeutic option for the treatment of aggressive neuroblastoma and other cancers where copper drives growth.

Catechin is widely known as an antioxidant, however, in certain conditions, such as in the presence of high level of metal ions, it can induce reactive oxidative stress species by Fenton reaction [33]. In particular, chemical studies have demonstrated that Cu^2+^ converts catechin from an antioxidant to a pro-oxidant [34]. However, the pro-oxidant activity of catechin in neuroblastoma cells has never been associated with their intracellular copper levels. In this work we revealed that Dextran-Catechin generates oxidative stress in neuroblastoma cells with high intracellular copper levels, but not in non-malignant cells with low intracellular copper levels.

Tumour cells are usually under high levels of oxidative stress and a further increase of reactive oxidative species can induce cell death [35]. To survive oxidative stress, cancer cells adopt anti-oxidant strategies, which protect against oxidative stress and can confer drug resistance [36]. Glutathione (GSH) plays a major role in the maintenance of the intracellular redox balance, and is involved in a number of metabolic processes and drug resistance [28, 37]. Importantly, GSH is considered the main intracellular copper binding agent regulating copper uptake in cells [27]. The binding of copper with GSH, limits the redox reaction of this metal ion with pro-oxidant agents. GSH is thus a key component that regulates intracellular copper levels and protects cancer cells from oxidative stress. Our results showed that with increasing concentrations of Dextran-Catechin there was a concomitant reduction in the levels of GSH. Therefore, Dextran-Catechin, not only induces oxidative stress by reacting with copper in neuroblastoma cells, but also deregulates the intracellular redox balance maintenance and copper homeostasis by reducing the levels of GSH.

**Figure 7:**
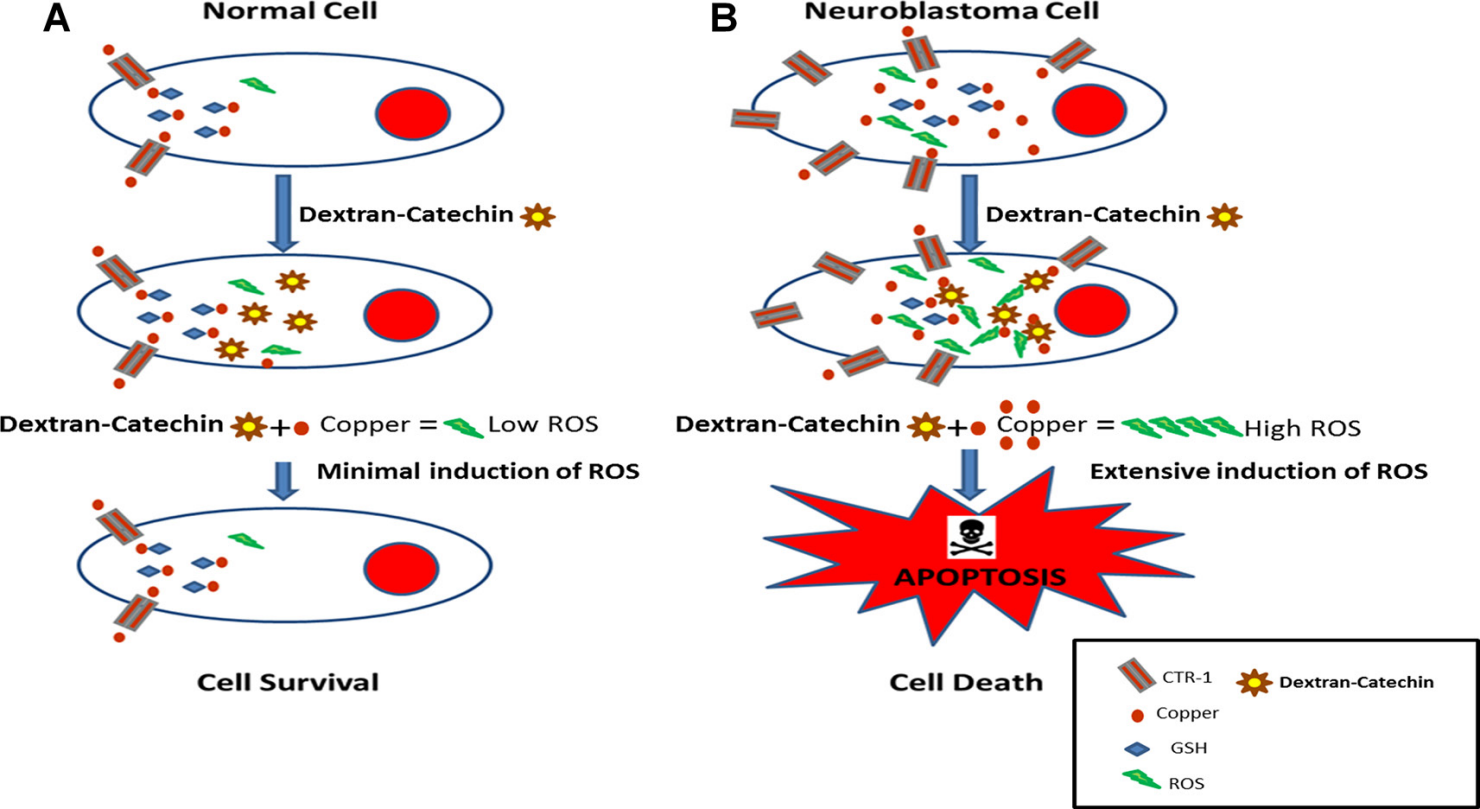
Schematic model of the proposed mechanism of action of Dextran-Catechin in cancer cells (**A**) Dextran-Catechin has a negligible effect in non-malignant cells that have low intracellular copper levels. (**B**) Dextran-Catechin induces oxidative stress and apoptosis in neuroblastoma cells with high levels of intracellular copper.

Copper homeostasis is a finely regulated process and unbalanced intracellular copper levels are associated with different pathologies. High copper content has been reported in a wide spectrum of adult cancers including breast, cervical, ovarian, lung, prostate, stomach, reticulo-endothelial system and leukemia [38]. Recently, it was reported that copper plays a role in BRAF signalling and tumorigenesis in melanoma, demonstrating the importance of this metal ion in cancer progression [39]. This and other studies showing a strong link between copper and cancer have led to a recent clinical trial using a copper chelating agent for the treatment of breast cancer at high risk of relapse, with encouraging preliminary results [40]. Although copper represents an interesting target for the development of anticancer drugs, physiological levels of copper are important for metabolism in normal cells [41]. In fact, chelation therapy may produce strong toxic effects, including kidney damage, irregular heartbeat, impairment of the immune system and neurological disorders [42]. What is unique about Dextran-Catechin is that its anticancer activity is not due to copper chelation but instead the intracellular reaction of this conjugate with copper, the levels of which are frequently elevated in cancer cells, which in turn generates oxidative stress and cell death (Figure 7). This represents an intriguing way to target the elevated copper levels in cancer cells in a range of cancers with less risk of side effects compared with the use of copper chelating agents. The robust anticancer activity of Dextran-Catechin, combined with its low toxicity in normal cells, and its novel mechanism of action make this conjugate a potential drug candidate for treating neuroblastoma and theoretically other cancers that have high intracellular copper.

## MATERIALS AND METHODS

### Cell culture

IMR-32 and SH-SY5Y cells were obtained from the ATCC; BE(2)-C and BE(2)-C/ADR were obtained from the late Prof June Biedler's lab. Upon receipt from ATCC, master stocks were prepared and cells for experiments were passaged for less than 6 months. IMR-32, SH-SY5Y and BE(2)-C master stock cells were validated by PCR (STR profiling) and stored in the Children's Cancer Institute Cell Bank.

IMR-32 cisplatin resistance cells were selected in escalating concentrations of cisplatin and the resultant cell line was designated IMR-32CisRes (Supplementary Figure 2B). All neuroblastoma cells were grown in DMEM medium supplemented with 10% FBS, and 1% L-glutamine. Normal lung fibroblasts, MRC-5, were obtained from ATCC and cultured as previously described [43]. Cell lines were grown as monolayers in a humidified atmosphere at 37°C and in 5% CO_2_. All cell lines were tested negative for mycoplasma contamination using the MycoAlert MycoPlasma Detection Kit (Lonza, Switzerland).

### Compounds

Dextran-Catechin was synthesised and characterised as previously described [10]. In particular, for the synthesis of this conjugate we used (+)-Catechin hydrate and Dextran 6000kDa (Sigma Aldrich, NSW, Australia). With a synthesis protocol optimized by our group [10], we obtained a conjugation of 19.9 mg of Catechin per g of Dextran-Catechin. A stock solution of Dextran-Catechin 1 mg/ml (1mg/ml refers to the concentration of catechin in the compound) was prepared in cell culture media DMEM and stored at −20°C. In order to have a direct comparison with the free Catechin, the concentrations of Dextran-Catechin used throughout the paper refers to the concentration of Catechin in the conjugate. Clinical grade cisplatin (10 mM) (Hospira Pty Limited, Melbourne, VIC, Australia) was stored at room temperature. CuCl_2_ and the copper chelator tetraethylenepentamine (TEPA) were prepared at a concentration of 1M in Milli-Q water (Sigma Aldrich, Castle Hill, NSW, Australia) and stored at −20°C.

### Cell viability assay

Cells were plated in clear transparent 96-well plates at optimized cell densities of 10^4^ cells/well for neuroblastoma cells and 5–10 × 10^3^ cells/well for MRC-5 cells. Treatment started after 24 or 48 hours, dependent on the cell line, to allow for cell attachment. Cells were either treated with Dextran-Catechin or Cisplatin, or the combination of Dextran-Catechin with CuCl_2_ and/or TEPA. Treatment effects on cell viability were determined based on the metabolic activity of cells using the Alamar Blue assay and spectrophotometric analysis as previously described [44]. All the cell viability assays have been conducted in the presence of 10% serum.

### Transfection of siRNA to knock down *CTR1*

BE(2)-C and IMR-32 cells were plated into 6-well plates at a density of 2 × 10^5^ and 5 × 10^5^ cells/well, respectively. Cells were transfected for 6 hours with two different siRNAs (Origene, Rockville, MD, USA) specific for *CTR1*, or a scrambled non-silencing siRNA, using Lipofectamine LTX reagent (Invitrogen, Carlsbad, CA, USA). Expression levels of *CTR1* mRNA and protein were monitored for 24 to 72 hours post-transfection using RT-qPCR and western blot analysis.

### Determining *CTR1* mRNA levels by Real-time quantitative PCR (RT-qPCR)

Total RNA was extracted by RNA isolation kit (Qiagen, Hilden, Germany) and cDNA generated with cDNA Synthesis Kits (Thermo Fisher Scientific, Victoria, Australia). For RT-qPCR, Power SYBR green PCR master mix (Applied Biosystems, Warrington, UK), forward and reverse primers for *CTR1* (Sigma, Castle Hill, NSW, Australia) and *GAPDH* primers (QuantiTect Primer Assays, Qiagen, Hilden, Germany) were used. The *CTR1* forward and reverse primer sequences were TCTTAGGTGCCGTCTCTA and AGTGGATACAGTGAGGATTC, respectively. RT-qPCR was performed with an Applied Biosystems 7900HT Fast Real-time PCR System. All procedures were conducted according to manufacturers' instructions. *CTR1* expression was normalized to that of the housekeeping gene *GAPDH*.

### Western blot

Protein electrophoresis of whole cell lysates and transfer onto PVDF membrane was performed as previously described [45]. Membranes were incubated with 10% skim milk powder in TBST (10 mM Tris, pH 8.0, 150 mM NaCl, 0.5% Tween 20) for 60 min, prior to washing once with TBST and incubating with antibodies against CTR1 [46] (rabbit anti-SLC31A1, ab129067, Abcam, Melbourne, VIC, Australia), PARP (rabbit anti-PARP antibody 9542, Cell Signaling Technology, Danvers MA, USA), or GAPDH (mouse anti-GAPDH, Abcam, Cambridge, UK). Membranes were washed and incubated with anti-rabbit or anti-mouse dilution of horseradish peroxidase-conjugated anti-antibody for 60 min. Blots were developed with Amersham ECL Prime Western Blotting Detection Reagent (GE Healthcare Life Sciences, Rydalmere, Australia) and analysed using QuanityOne (Version 4.6.9, Bio-Rad, Gladesville, Australia). Both CTR1 and PARP protein expression were normalized to that of the housekeeping protein GAPDH.

### Measurement of intracellular copper

IMR-32, IMR32-CisRes, BE(2)-C and MRC-5 cells were grown in cell culture dishes to 70% confluence. Following three washes in PBS, they were scraped in Milli-Q water supplied with protease inhibitor (Sigma Aldrich, Castle Hill, NSW). Intracellular copper levels were measured using the QuantiChrom^™^ Copper Assay Kit (BioAssay Systems, Hayward, CA, USA). Copper levels were determined according to manufacturer's instructions by absorbance spectrophotometry and normalized to cell protein content.

### Quantification of combination effect

The Excess Over Highest Single Agent (EOHSA) model was used to quantify the effect of combination treatment. The EOHSA is the difference in cell viability between the combination treatment and the most effective single compound at the corresponding concentration [47]. The EOHSA was visualized in surface plots. Compound combinations which exceeded the highest effect of the single compounds at corresponding concentration are represented by positive values, while negative values indicated that the effect induced by a single compound was greater than that of the combination.

### Trypan blue exclusion assay

BE(2)-C and IMR-32 cells were plated at a density of 2 × 10^5^ and 5 × 10^5^ cells/well, respectively, into 6-well plates. Twelve hours post-transfection with two different *CTR1* specific siRNAs (siRNA A and siRNA B Origene, Rockville, MD, USA) or scrambled non-silencing siRNA, cells were treated with Dextran-Catechin for 24 hours. Cells were harvested using trypsin. Cell number and viability were quantified by staining with 0.4% trypan blue solution (Life Techologies, Mulgrave, Vic, Australia), which allowed counting of viable and non-viable cells using a haemocytometer.

### Oxidative stress detection

To assess oxidative stress, a fluorogenic probe (CellROX^®^ Green Reagent, Life Technologies, Vic Australia) designed to reliably measure reactive oxygen species (ROS) in live cells was used. The cell-permeable reagents are non-fluorescent while in a reduced state and upon oxidation exhibit a strong fluorogenic signal. IMR-32, IMR-32-CisRes and MRC-5 cells were plated in black 96-well plates with transparent bottoms at 10^4^ cells/well. Next day, cells were incubated for 5 h with Dextran-Catechin, prior to washing in PBS and measuring oxidative stress according to manufacturer's instructions using a fluorometer (Wallac VICTOR, PerkinElmer, Massachusetts, USA).

### Fluorescence lifetime imaging (FLIM) microscopy: data acquisition

The phasor approach to fluorescence lifetime imaging microscopy (FLIM) was used to assess the intracellular reduction-oxidation ratio by measuring free and bound NADH. FLIM data were acquired with the Leica TCS SP5 laser scanning microscope, coupled to a 2-photon Ti:Sapphire laser (Spectra-Physics Mai Tai, Newport Beach) producing 80 fs pulses at a repetition of 80 MHz, and a Picoquant Microtime200 to acquire the lifetime data. A 63× oil immersion objective 1.4 N.A. was used for all experiments.

Cellular auto-fluorescence was excited at 740 nm with the 2-photon laser. A SP 690 nm dichroic filter was used to separate the fluorescence signal from the laser light. The fluorescence signal was detected by a SPAD detector that had a 555LP filter placed in front. For image acquisition, the pixel frame size was set to 256 × 256 and scan rate to 400 Hz, with a line average of 8. The average laser power at the sample was maintained at the mW level. The FLIM data were acquired with the MT200 software developed by Dr. Ales Benda and the phasor transformation and data analysis processed by the SimFCS software developed at Laboratory for Fluorescence Dynamics (www.lfd.uci.edu). Calibration of the system and phasor plot space was performed by measuring Atto425, which has a known single exponential lifetime of 3.6 ns. IMR-32 and MRC-5 cells were plated at a density of 5 × 10^4^ cells/well into confocal 35-mm glass-bottom Petri dishes. Cells were imaged on a microscope equipped with a stage-top incubator and CO_2_ controller. Live imaging of cells was performed before and after 1 h and 3 h of Dextran-Catechin treatment. FLIM data were acquired with the Leica TCS SP5 laser scanning microscope, coupled to a 2-photon Ti:Sapphire laser (Spectra-Physics Mai Tai, Newport Beach) producing 80 fs pulses at a repetition of 80 MHz, and a Picoquant Microtime200 to acquire the lifetime data. A 63× oil immersion objective 1.4 N.A. was used for all experiments.

### Fluorescence lifetime imaging microscopy: data analysis

The phasor approach to fluorescence lifetime imaging microscopy was employed to analyse changes in cellular auto-fluorescence. This method transforms the fluorescence decay in each pixel of a FLIM image into the sine and cosine components, which are then represented in a two dimensional polar plot (phasor plot). Each pixel of the FLIM image gives rise to a single point (phasor) in the phasor plot and when used in reciprocal mode, enables each point of the phasor plot to be mapped to each pixel of the FLIM image. Since phasors follow simple vector algebra, it is possible to determine the fractional contribution of two or more independent molecular species coexisting in the same pixel. Thus in the case of two independent species, all possible weightings give a phasor distribution along a linear trajectory that joins the phasors of the individual species in pure form. In the case of auto-fluorescence we detect a linear distribution that extends from 0–100% bound NADH / free NADH. By moving the phasor cursor along the straight line drawn between these two terminal phasor locations, we can calculate the exact fractional contribution of free NADH for each pixel highlighted.

### Measurement of cellular GSH

IMR-32 cells were plated at a density of 5 × 10^5^ cells/well into 6-well plates. L-BSO (Santa Cruz Biotechnology, Santa Cruz, CA) was used as a positive control that reduces cytoplasmic GSH levels. Addition of L-BSO from 5–100 mM to cell culture media caused no toxicity after 24 hours, and 10 mM L-BSO was subsequently used. To assess changes in intracellular GSH, cells were treated with concentrations between 10–45 mg/ml Dextran-Catechin for 3 hours. GSH levels were determined with the Glutathione Assay Kit (Sigma Aldrich, Castle Hill, NSW, Australia).

### Neuroblastoma xenograft and syngeneic mouse models: Dextran-catechin treatment

Animal studies were approved by the Animal Ethics Committee at UNSW Australia (AEC# 14/36B) and animals were either bred on site (TH-MYCN +/+) or obtained from the Australian Bio Resources Facility (Moss Vale, NSW, Australia).

Female BALB/c-Fox1nu/Ausb and Balb/c mice, 6–8 weeks old were injected subcutaneously into the right flank with 4 × 10^6^ human IMR-32 cells (BALB/c-Fox1nu/Ausb) or mouse NHO2A cells (Balb/c) suspended in 100 μL PBS and growth factor–reduced Matrigel (BD Biosciences) at a 1:1 ratio. When tumours reached approximately 200 mm^3^, mice were randomised into treatment groups (4 mice per group) and treated once per week for a total of 3 weeks with 0, 75, 150 or 300 μg/ml of Dextran-Catechin. Tumour size was measured using calipers and mice were euthanized once tumours reached 1,000 mm^3^. Tumour volume was calculated using the formula ½ × L × W^2^, where L and W represent the longer and shorter dimension, respectively. Tumour tissue was collected post-sacrifice and placed into 4% paraformaldehyde for histologic analysis or snap frozen in liquid N_2_ for biochemical analysis.

### Statistical analysis

Data are presented as mean +/− SEM of at least three independent experiments. Multiple statistical comparisons between different groups were performed by using one-way ANOVA (GraphPad Prism 5, GraphPad Software Inc., La Jolla, CA).

## SUPPLEMENTARY MATERIALS


