# Supplementary material for: Dextran-Catechin: An anticancer chemically-modified natural compound targeting copper that attenuates neuroblastoma growth
Source: Oncotarget. 2016 Jun 21;7(30):47479–93. doi: 10.18632/oncotarget.10201 (PMC5216955; doi:10.18632/oncotarget.10201)
Supplement: Supplementary file 1 [file oncotarget-07-47479-s001.pdf]

Dextran-Catechin: An anticancer chemically-modified natural compound targeting copper that attenuates neuroblastoma growth

Supplementary Materials

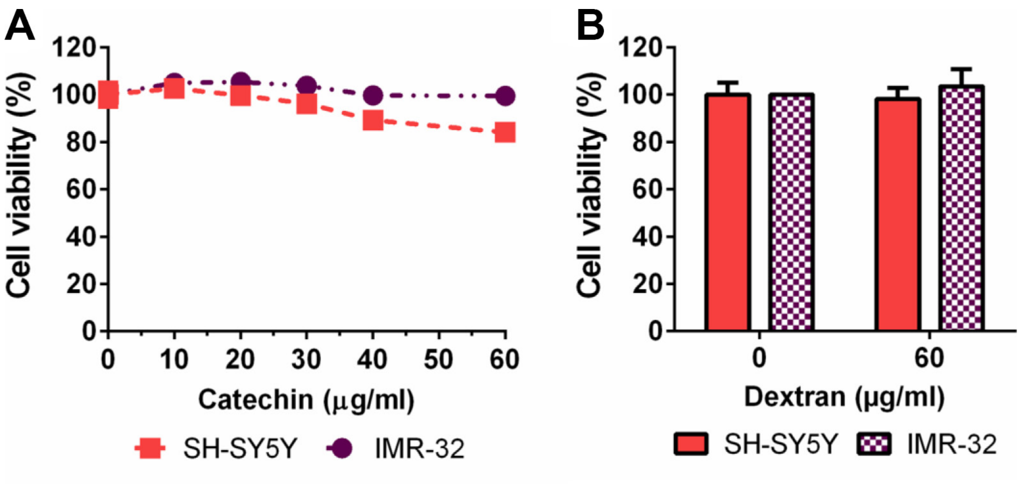

Supplementary Figure S1: Effect of free Catechin and Dextran on cell viability of neuroblastoma cells. SH-SY5Y and IMR-32 cells were treated with (**A**) 0–60 µg/ml Catechin or (**B**) 60 µg/ml Dextran for 72 h and cell viability was measured using Alamar blue.

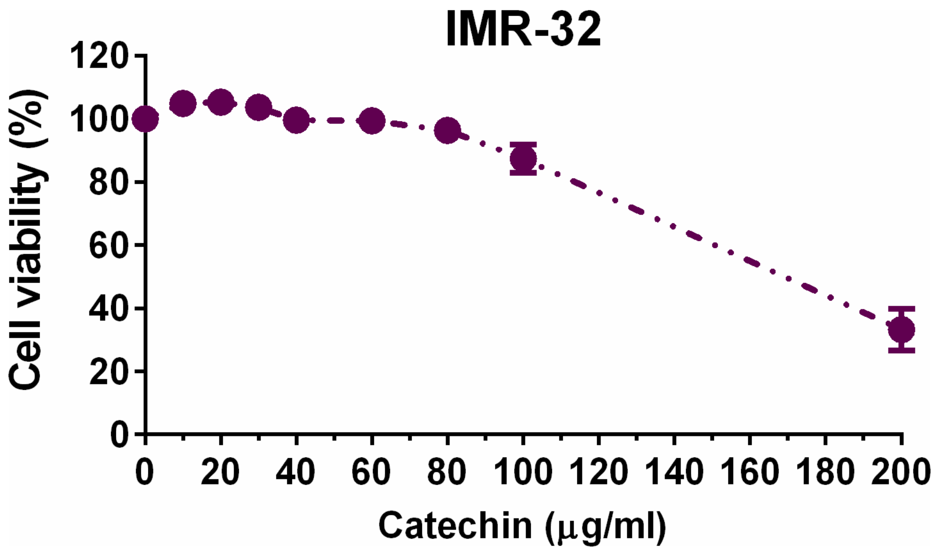

Supplementary Figure S2: Determination of IC_{50} for free Catechin in neuroblastoma cells. IMR-32 cells were treated with escalating doses of 0–200 µg/ml Catechin for 72 h and cell viability was measured using Alamar blue. The IC_{50} for Catechin resulted 170 ± 3.84 µg/ml.

Supplementary Data 3: Ferric reducing ability of plasma (FRAP) assay to test the stability of Catechin and Dextran-Catechin in presence of serum. The improved serum stability of Dextran-Catechin was proved by determining the ferric reducing ability (FRAP assay) [1, 2]. The initial FRAP value for free Catechin was 629.2 ± 24.0 µM TE (0 h), decreasing to 254.2 ± 21.0 µM TE after 72 h [3, 4]. Dextran Catechin showed a similar FRAP value (651 ± 31.4 µM TE) for 0 h, which remained almost unchanged over the reaction time (604 ± 27.3 µM TE at 72 h). The FRAP assay was performed according to the literature with minor modifications [5]. Fresh FRAP reagent was prepared in acetate buffer (25.0 ml, 300 mM, pH 3.6) by mixing 2,4,6-Tris (2-pyridyl)-S-triazine solution (2.5 ml, 10 mM in 40 mM HCl) and $FeCl_3 \cdot 6H_2O$ solution (2.5 ml, 20 mM). The reagent was warmed to 37°C and the absorbance (1.5 mL) read. 50 µL of each sample was added and absorbance was measured after 20 min at a wavelength of 593 nm using a Jasco V-530 UV-Vis spectrometer (Jasco Europe, Milan, Italy). Trolox (1 to 100 µM) was chosen as a standard antioxidant and activity of samples (500 µM equal concentration) was expressed as Trolox equivalents (µM TE).

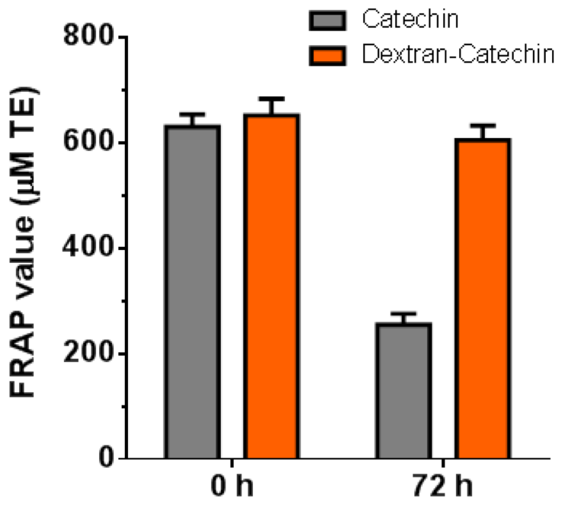

Supplementary Figure S3: FRAP values for free Catechin and Dextran-Catechin in cell culture media supplemented with 10% of FBS serum.

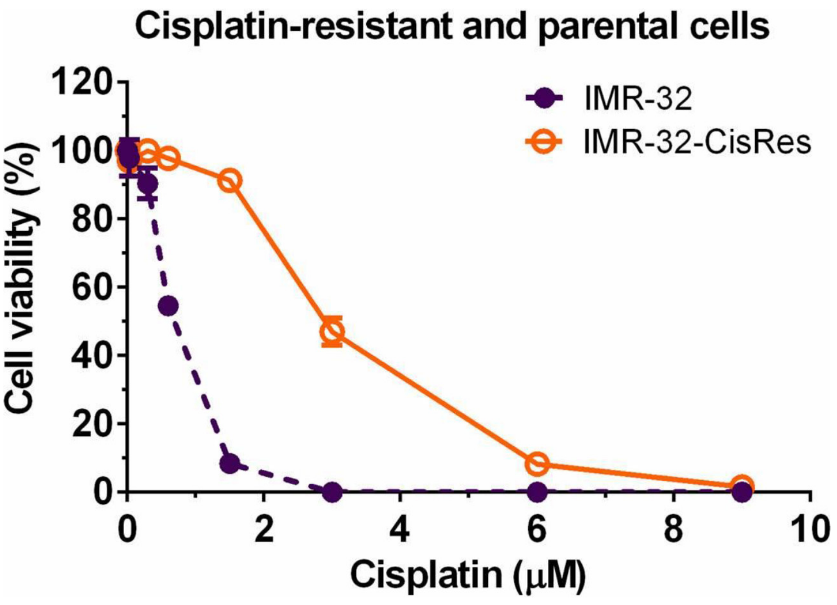

Supplementary Figure S4: IMR-32 cells selected for resistance to cisplatin. IMR-32 cisplatin resistant cells were selected in escalating concentrations of cisplatin as follows: parental IMR-32 cells were treated for 3 days with the ID90 concentration of cisplatin (1.1 µM) and surviving cells were allowed 3 weeks to recover, then re-treated. This process continued until treatment no longer affected cell growth (4 treatments in total). Cisplatin concentration was increased, firstly to 2.2 µM (7 treatments) then to 3.3 µM (6 treatments). At this point selection ceased and the cell line was designated IMR-32CisRes. IMR-32 cells and IMR-32-CisRes had similar cell doubling times (62 and 66 h, respectively). Cell viability assays showed that the IMR-32-CisRes cells were resistant to cisplatin (IC_{50} 2.96 ± 0.15 µM) compared to the parental IMR-32 cells (IC_{50} 0.685 ± 0.0346 µM) after 72 h treatment. Values are calculated as mean of at least three individual experiments.

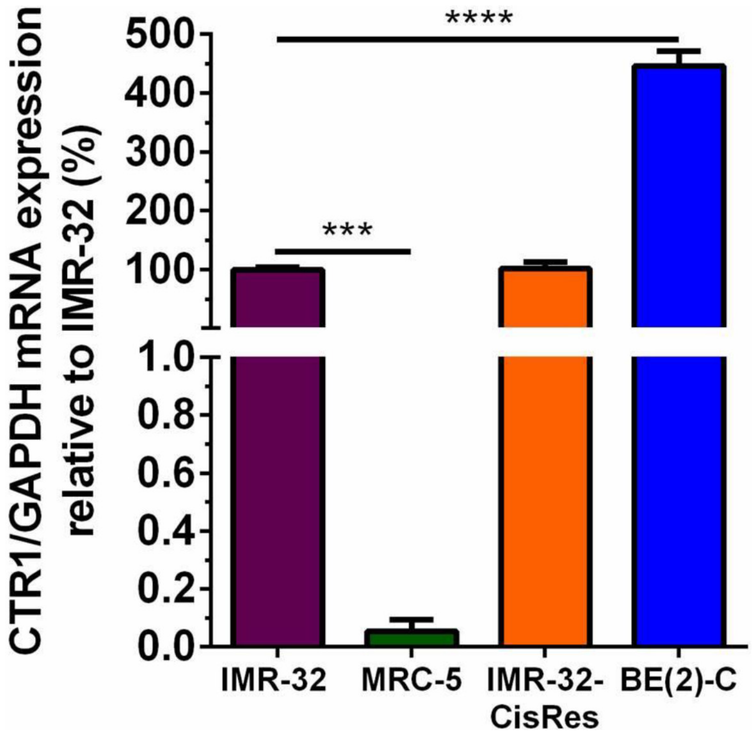

Supplementary Figure S5: Expression of *Ctr1* mRNA is significantly higher in neuroblastoma cells compared to non-malignant fibroblasts. *Ctr1* mRNA expression was assessed in IMR-32, IMR-32-CisRes, BE(2)-C and MRC-5 cells, normalized to GAPDH gene expression and is shown relative to the expression in IMR-32 (100%). *Columns*, means of at least three individual experiments; *Bars*, SEM (***$p < 0.001$, ****$p < 0.0001$).

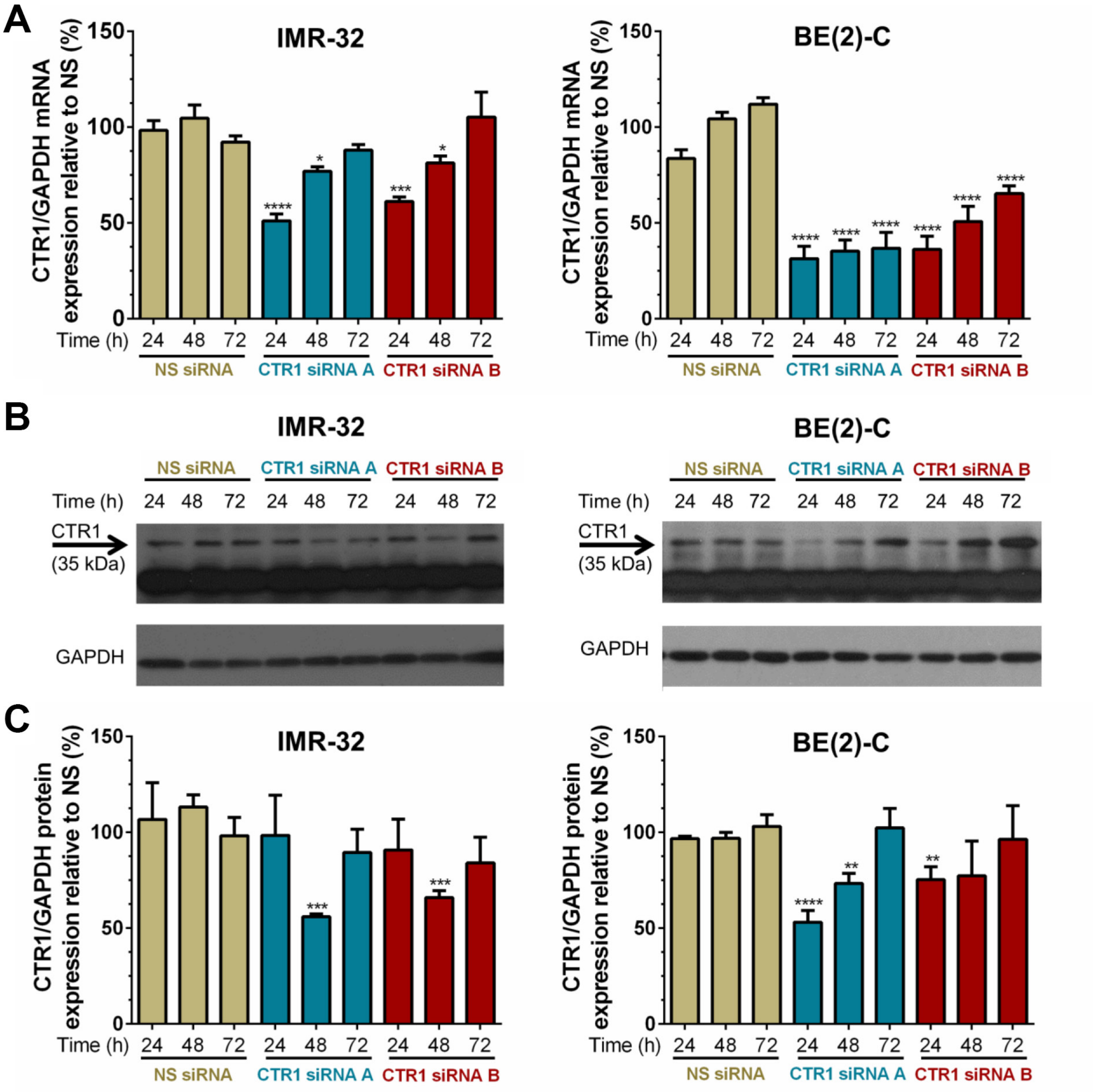

Supplementary Figure S6: Knockdown using siRNA significantly reduced *Ctr1* mRNA and protein expression in IMR-32 and BE(2)-C. To reduce CTR1 levels, specific siRNA was used at concentrations of 40 and 20 nM in IMR-32 and BE(2)-C cells, respectively, and mRNA and protein expression was monitored over 24–72 h. (**A**) *Ctr1* mRNA expression in IMR-32 and BE(2)-C cells normalised to that of GAPDH. (**B**) Representative western blots of CTR1 protein in IMR-32 and BE(2)-C cells. GAPDH expression was used as protein loading control. (**C**) Densitometry graph of Western blots of CTR1 protein expression. *Colums*, means of at least three individual experiments; *Bars*, SEM (*$p < 0.05$, **$p < 0.01$, ***$p < 0.001$, ****$p < 0.0001$).

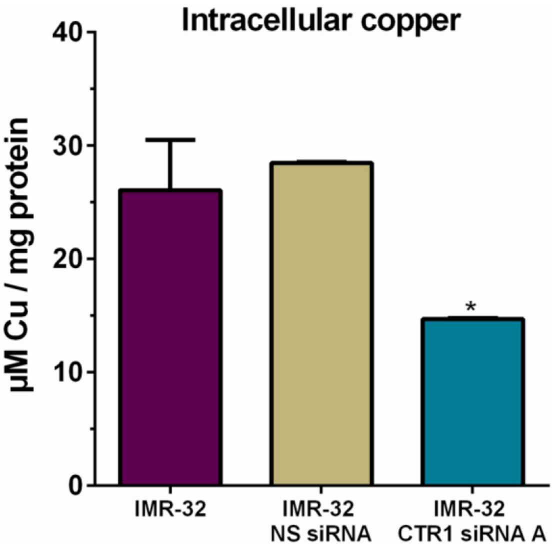

Supplementary Figure S7: Depletion of CTR1 reduced the intracellular copper concentration in neuroblastoma cells. IMR-32 cells were transfected with a CTR1 specific siRNA A (CTR1 siRNA A) or a control non-silencing siRNA (NS siRNA) at concentrations of 40 nM. Measurements of intracellular copper levels were performed by spectrophotometric analysis 24 h after siRNA transfection. Depletion of CTR1 results in decreased intracellular copper expressed as μM Cu / mg protein. Bars, SEM (**p* < 0.05).

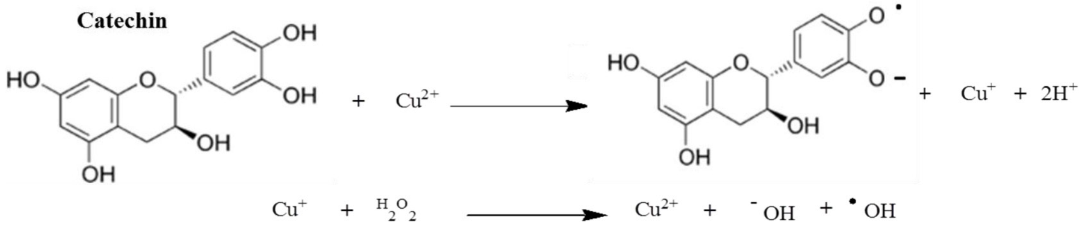

Supplementary Data S8: Dextran-Catechin reacts with Copper by Fenton reaction and induces the production of reactive oxidative species. Phenolic compounds, and tea catechins in particular, are antioxidant agents due to their reducing properties, but, in the presence of metal ions, such as cupric ions, they can switch to pro-oxidant compounds with relevant biological implications [6, 7], according to the following reaction: (Equation 1). $Cu2^+$ acts as a one-electron oxidant and it is reduced to C^{u+} by catechin moieties [8]. The re-oxidation of Cu^+ to $Cu2^+$ by reaction with H_2O_2 is accompanied by the formation of ROS [6]. Here, to prove the Fenton reaction between copper and Dextran-Catechin, we measured the disappearance of H_2O_2 in the presence of $Cu(Cl)_2$ 10 and 100 μM and Dextran-Catechin 20 μg/ml by using a specific luminescent probe to specifically detect H_2O_2 (ROS-Glo™ H_2O_2 assay, Promega USA). The results show that Dextran-Catechin 20 μg/ml in presence of $Cu(Cl)_2$ 10 and 100 μM reduced H_2O_2 of 36% and 57 % respectively.

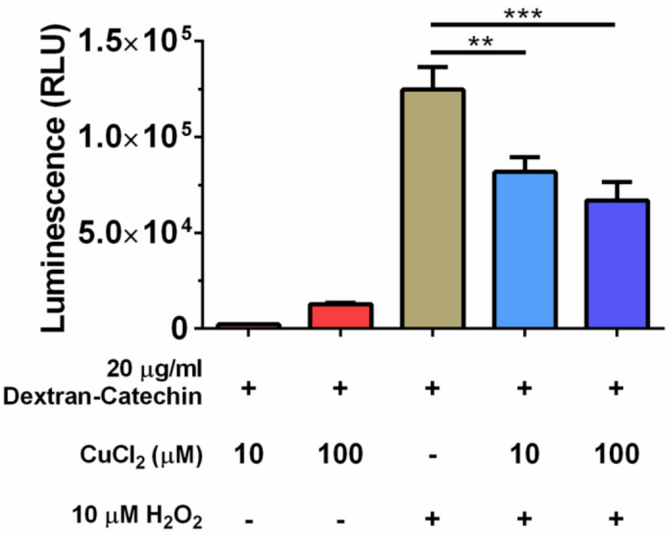

Supplementary Figure S8: Dextran-Catechin reacts with copper reducing the amount of H_2O_2. According to equation 1 (Fenton reaction), we detected a decrease of H_2O_2 after combination with Dextran-Catechin and $Cu(Cl)_2$ using a luminescent probe. Compared to 20 µg/ml Dextran-Catechin alone, H_2O_2 was reduced to 36% and 57 % after adding 10 and 100 µM $Cu(Cl)_2$ respectively. *Columns*, means of at least three individual experiments; *Bars*, SEM (***$p < 0.001$, **$p < 0.01$).

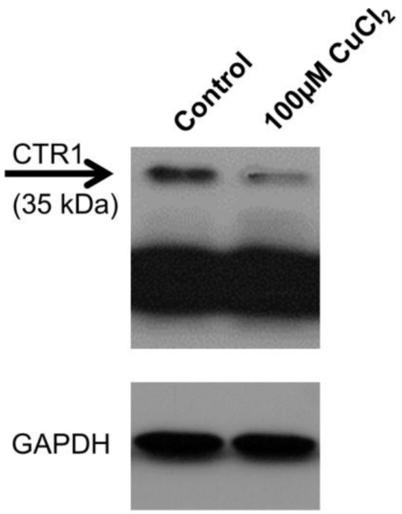

Supplementary Figure S9: Supplementation of copper in the culture media induced down-regulation of CTR1 in IMR-32 neuroblastoma cells. Representative western blots of CTR1 protein in IMR-32 (control) before and after 12 h of incubation with 100 µM $Cu(Cl)_2$.

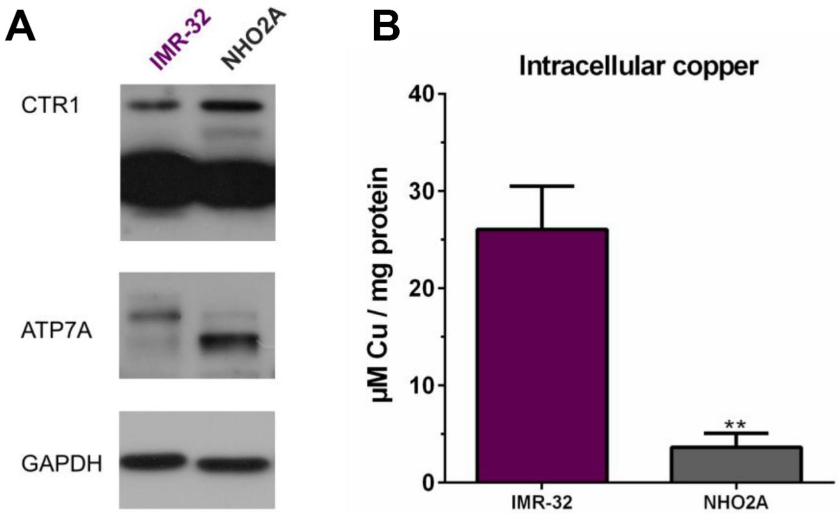

Supplementary Figure S10: Expression levels of CTR1 and ATP7A in human IMR-32 compared to murine NHO2A neuroblastoma cells. (**A**) CTR1 is highly expressed in both human and murine neuroblastoma cell lines; however, the copper efflux protein ATP7A is higher in the murine NHO2A neuroblastoma cells; (**B**) Intracellular copper levels in NHO2A (0.685 ± 0.0346 µM) are lower compared to that in IMR-32 cells.

REFERENCES

1. Alam MN, Bristi NJ, Rafiquzzaman M. Review on in vivo and *in vitro* methods evaluation of antioxidant activity. Saudi Pharmaceutical Journal 2013; 21:143–152
2. Benzie IFF, Strain JJ. The ferric reducing ability of plasma (FRAP) as a measure of 'antioxidant power': The FRAP assay. Analytical Biochemistry 1996; 239:70–76.
3. Skroza D, Generalić Mekinić I, Svilović S, Šimat V, Katalinić V. Investigation of the potential synergistic effect of resveratrol with other phenolic compounds: A case of binary phenolic mixtures. Journal of Food Composition and Analysis. 2015; 38:13–18.
4. Dube A, Ng K, Nicolazzo JA, Larson I. Effective use of reducing agents and nanoparticle encapsulation in stabilizing catechins in alkaline solution. Food Chemistry 2010; 122:662–667.
5. Barreca D, Laganà G, Leuzzi U, Smeriglio A, Trombetta D, Bellocco E. Evaluation of the nutraceutical, antioxidant and cytoprotective properties of ripe pistachio (Pistacia vera L., variety Bronte) hulls. Food Chemistry 2016; 196:493–502.
6. Iwasaki Y, Hirasawa T, Maruyama Y, Ishii Y, Ito R, Saito K, Umemura T, Nishikawa A, Nakazawa, H. Effect of interaction between phenolic compounds and copper ion on antioxidant and pro-oxidant activities. Toxicology in Vitro 2011; 25:1320–1327.
7. Hayakawa F, Kimura T, Maeda T, Fujita M, Sohmiya H, Fujii M, Ando T. DNA cleavage reaction and linoleic acid peroxidation induced by tea catechinsin the presence of cupric ion. Biochimica et Biophysica Acta 1997; 1336:123–131.
8. Mochizuki M, Yamazaki S, Kano K, Ikeda T. Kinetic analysis and mechanistic aspects of autoxidation of catechins. Biochimica et Biophysica Acta 2002; 1569:35–44.
